# Microvascular Function, Inflammatory Status, and Oxidative Stress in Post-Bariatric Patients with Weight Regain

**DOI:** 10.3390/nu15092135

**Published:** 2023-04-29

**Authors:** Karynne Grutter Lopes, Maria das Graças Coelho de Souza, Eliete Bouskela, Luiz Guilherme Kraemer-Aguiar

**Affiliations:** 1 Obesity Unit, Centro de Pesquisas Clínicas Multiusuário (CePeM), Hospital Universitário Pedro Ernesto (HUPE), State University of Rio de Janeiro, Rio de Janeiro 20550-013, RJ, Brazil; mgcsouza@gmail.com (M.d.G.C.d.S.); eliete.bouskela@gmail.com (E.B.); lgkraemeraguiar@gmail.com (L.G.K.-A.); 2 Postgraduate Program in Clinical and Experimental Physiopathology (Fisclinex), Faculty of Medical Sciences, State University of Rio de Janeiro, Rio de Janeiro 20550-013, RJ, Brazil; 3 Laboratory for Clinical and Experimental Research on Vascular Biology (Biovasc), Rio de Janeiro State University, Rio de Janeiro 20550-013, RJ, Brazil; 4 Endocrinology, Department of Internal Medicine, Faculty of Medical Sciences, State University of Rio de Janeiro, Rio de Janeiro 20550-013, RJ, Brazil

**Keywords:** bariatric surgery, weight regain, microvascular function, chronic inflammation

## Abstract

Weight loss after bariatric surgery in obesity improves vascular function and metabolic/inflammatory profiles and reduces cardiovascular mortality but there are limited data on the effects of weight regain on vascular health. We compared the metabolic/inflammatory profiles, oxidative status, and vascular function of post-bariatric patients with a high ratio of weight regain (RWR) vs. non-surgical controls. Thirty-two post-bariatric patients [Roux-en-Y gastric bypass; aged = 44 ± 8 years, BMI = 40.1 ± 7.7 kg/m^2^, and RWR = 58.7 ± 24.3%] and thirty controls that were BMI-, age-, and gender-matched entered the study. We collected clinical data, metabolic/inflammatory/oxidative stress circulating biomarkers, and endothelial/microvascular reactivity through Venous occlusion plethysmography and Laser speckle contrast imaging. The bariatric group exhibited lower neck circumference, fasting glucose, and triglycerides than the non-surgical group, while HDL-cholesterol was higher in the bariatric group (*p* < 0.001). There was no significant difference between groups for endothelial/microvascular reactivities (*p* ≥ 0.06). Resistin, leptin, endothelin-1, soluble forms of intercellular cell adhesion molecule-1 and vascular cell adhesion molecule-1, tumor necrosis factor-α, and thiobarbituric acid reactive substances did not differ significantly between groups (*p* ≥ 0.09) either. The adiponectin level was higher in the bariatric compared to the non-surgical group, while interleukin-6 was lower in the bariatric group (*p* < 0.001). Despite the fact that endothelial/microvascular functions were not significantly different between groups, post-bariatric patients present partially preserved metabolic/inflammatory benefits even with high RWR.

## 1. Introduction

Surgical treatment of obesity promotes substantial weight loss, improves metabolic profile, and induces resolution or improvement of obesity-related comorbidities, including dyslipidemia, type 2 diabetes mellitus (T2D), hypertension, and sleep disorders [1,2]. All these have a positive effect on cardiovascular risk [1,3,4,5,6] and all-cause mortality while simultaneously lowering long-term healthcare costs [7,8].

Bariatric surgery is associated with improved endothelium-dependent vasodilatation in patients with obesity compared to clinical treatment [9,10,11]. It is also associated with a 65% reduction in major macrovascular and microvascular events in patients with obesity and T2D [6]. This is relevant given the relationship between endothelial dysfunction and atherosclerosis, which reflects mainly the reduction of nitric oxide availability that is in the core of the pathophysiology of the disease [12]. These favorable effects on vascular function were associated with weight loss and improvements in metabolic profile [9], with an attenuation of oxidative stress and on levels of systemic inflammatory biomarkers [13,14].

Surgical treatment of obesity covers a wide range of bariatric procedures. One of the most employed is the Roux-en-Y gastric bypass. This procedure has high efficacy rates in reducing body mass, but weight regain may also occur in some patients. This undesirable outcome may lead to significant health consequences, including the recurrence of cardiometabolic comorbidities [15]. On a pathophysiological basis, regaining weight may negatively affect vascular function by losing vasculoprotective factors through metabolic dysregulation, oxidative stress, low-grade systemic inflammation, and endothelial dysfunction [13]. It is known that a healthy endothelium is responsible for vascular homeostasis, maintaining the balance between vasodilators/vasoconstrictors agents and pro- and anticoagulant factors [12]. 

Specifically, in obesity, dysfunctional adipose tissue predominantly secretes proinflammatory adipokines (e.g., leptin, resistin, interleukin-6 (IL-6), and tumor necrosis factor-α (TNF-α)) in detriment of the anti-inflammatory ones (e.g., adiponectin). This dysregulation elicits the activation of the endothelium, turning it into a proinflammatory and a procoagulant phenotype with increased production of reactive oxygen species (ROS), less bioavailability of nitric oxide, and augmented expression of vasoconstrictors, including endothelin-1 [16,17,18]. Previous studies performed in our laboratory have shown that obesity per se is sufficient to elicit an impairment of microvascular reactivity [19,20] and that body mass index (BMI) positively correlates with the worsening of forearm blood flow after intra-arterial administration of endothelium-dependent and -independent vasodilators [19]. Moreover, the observed decline of endothelium-dependent and -independent functions was accompanied by a significant increase in blood levels of leptin and IL-6 and by a significant decrease in circulating levels of adiponectin [19]. 

Despite the undeniable benefits of bariatric surgery on cardiovascular function [1,3,4,5,6], little is known about the effects of regaining weight after bariatric procedure on cardiovascular risk. To our knowledge, data concerning the underlying mechanisms associated with vascular function in this group with weight recurrence are limited to clinical variables, underscoring the need for further investigation. Concerning this gap in the literature, we compared the metabolic profile, inflammatory/oxidative status, and vascular function of patients who were subjected to bariatric surgery with a high ratio of weight regain (RWR) vs. non-surgical BMI-matched controls.

## 2. Materials and Methods

### 2.1. Subjects 

We recruited 289 individuals with obesity subjected to bariatric procedure at the outpatients’ care unit in the State University of Rio de Janeiro, Brazil. The inclusion criteria were patients having a Roux-en-Y gastric bypass (RYGB) surgery performed laparoscopically, with a mean weight loss following surgery of at least 50% of their initial body weight and a recovery of weight of more than 40% from the nadir weight. The exclusion criteria were individuals that underwent revisional surgery or any other bariatric procedure, pregnancy, smoking, alcoholism, physical activity ≥ 150 min/week, recent coronary syndromes, including heart failure and myocardial infarction, stroke, malignant neoplasm, or systemic infection. 

Of those 289 patients, 136 were not available to participate. One was pregnant, 1 had recent breast cancer, 1 had rheumatic diseases, 1 had insufficient weight loss after surgery, 2 had alcoholism, 4 were smokers, 8 were physically active, 25 were submitted to other bariatric procedures, and 78 of them had weight regain but less than 50%. As result, a total of 32 post-bariatric patients [28 females (87.5%), aged = 44 ± 8 years and BMI = 40.1 ± 7.7 kg/m^2^] (bariatric group) and 30 BMI-, age-, and gender-matched controls (non-surgical group) participated in the study.

Hypertension, T2D, and dyslipidemia were defined by blood pressure ≥ 130 or 80 mmHg and/or in use of antihypertensive drug [21], glycated hemoglobin (HbA1c) ≥ 6.5% or fasting plasma glucose ≥ 126 mg/dL or the use of antidiabetic drugs [22], and low-density lipoprotein cholesterol (LDL-cholesterol) ≥ 160 mg/dL, or triglyceride ≥ 150 mg/dL, or high-density lipoprotein cholesterol (HDL-cholesterol) < 40 and <50 mg/dL, for men and for women, respectively [23]. All patients who underwent surgeries outside our unit had the RYGB procedure posteriorly confirmed by digestive endoscopy.

### 2.2. Research Design

Recruitment, pre-participation screening, and data collection occurred between April 2021 and May 2022. All assessments occurred over two days, interspersed with one to two week intervals, in the following order: (a) first visit: patients were asked to provide their written informed consent, demographic characteristics, clinical history and to undergo a physical examination, and anthropometric measurements, and (b) second visit: blood samples collection to evaluate inflammatory, endothelial injury, and oxidative stress biomarkers, and to assess microvascular reactivity. Microvascular assessments were performed after 20 min of rest with patients in the supine position, after 8 h overnight fasting, in a quiet, temperature-controlled room (23 ± 1 °C), between 7 and 11 A.M. The patients were instructed to take their usual medications on the morning of the exams. The results of biochemical analyses were gathered from the individuals’ medical records.

### 2.3. Ethical Approval

This cross-sectional study was approved by the local ethics committee (CAAE: 16425419.8.0000.5259) and registered in ClinicalTrials.gov (NCT04193397). Participants were provided with a detailed explanation of the potential benefits and risks of the study before they signed the written informed consent. All procedures were performed according to the principles outlined in the Declaration of Helsinki. 

### 2.4. Anthropometric Measurement

Weight and height were measured on a standardized scale (Welmy^TM^ W300A, São Paulo, SP, Brazil). BMI was weight in kilograms divided by height in meters squared. Neck, waist, and hip circumferences were assessed by flexible measuring tape using standard procedures. Outcome surgical measures included: (a) preoperative BMI and nadir weight; (b) the percentage of excess weight loss (EWL), as (preoperative weight − nadir weight)/(preoperative weight − ideal weight) × 100%, and (c) the percentage of RWR, as (current weight − nadir weight)/(preoperative weight − nadir weight) × 100%. Patients’ ideal weight postoperative was defined by the weight divided by the height resulting in a BMI of 25 kg/m^2^ [24]. The preoperative and nadir weights were self-reported twice, once from values recorded in the patients’ medical records and others when they were invited to participate in the study. We also called all patients who self-reported their data six months after their first visit and asked them again about their preoperative and minimum postoperative weights.

### 2.5. Functional Assessment of Forearm Endothelial Reactivity by Venous Occlusion Plethysmography (VOP) 

Forearm blood flow was evaluated through venous occlusion plethysmography (Hokanson^TM^ AI6, Bellevue, WA, USA), as previously described [25]. VOP was performed in four sequential steps, as follows: forearm blood flow (FBF) at baseline 1; Peak FBF during reactive hyperemia which is the vasodilatory response after ischemia release (endothelium-dependent), after 5 min of forearm arterial occlusion with pressure 50 mmHg above systolic blood pressure; FBF at baseline 2; and Peak FBF after 5 min of 0.4 mg sublingual nitroglycerin administration (Nitrolingual Burns Adler Pharmaceuticals^TM^ Inc., Charlotte, NC, USA). Nitroglycerin is an exogeneous nitric oxide donor used to verify vascular wall integrity through endothelial-independent vasodilatation. The duration of the measurements was 2 min in each phase, followed by 3 min intervals, to avoid interference in the next phase, except between reactive hyperemia and FBF baseline 2, when a 15 min interval was applied. Heart rate was measured continuously by lead II ECG (Hokanson^TM^ RT2000, Bellevue, WA, USA). A semi-automated oscillometric device measured blood pressure before each phase (LifeWindow LW6000, Digicare Biomedical Technology^TM^, West Palm Beach, FL, USA). All assessments were carried out by a single trained technician (intra-observer measurement variation—8%). Inter-individual coefficients of variation for outcomes assessed using VOP ranged from 10–15%. 

### 2.6. Functional Assessment of Cutaneous Microvascular Reactivity Using Laser Speckle Contrast Imaging (LSCI)

Laser speckle contrast imaging evaluated microvascular reactivity at the cutaneous site with a laser wavelength of 785 nm (PeriCam PSI system, Perimed^TM^, Stockholm, Sweden). Continuous measurements of cutaneous microvascular reactivity in the forearm were performed using a 70-mW system and sampling rate of 18 Hz. Image acquisition and analysis were performed using PIM Soft software (Perimed^TM^, Stockholm, Sweden) according to the manufacturer’s instructions. Four steps were registered: Baseline flow, peak flow during post-occlusive reactive hyperemia (PORH), post-occlusive flow, and duration of PORH. Baseline measurements were acquired for 2 min, PORH was assessed after arterial occlusion with supra-systolic pressure (50 mmHg above systolic arterial pressure) using a sphygmomanometer applied to the right arm for 3 min. The skin reperfusion period was recorded after releasing the pressure for 5 min. The measurements of blood flow were divided by mean arterial pressure to calculate the cutaneous vascular conductance during baseline and PORH periods (in arbitrary perfusion units/mmHg) This assessment is used as marker of atherothrombotic disease and stratification of cardiovascular risk [26].

### 2.7. Biochemical and Hormonal Analysis 

Blood levels of HbA1c were evaluated using turbidimetric inhibition method. Plasma glucose was determined using the glucose oxidase colorimetric method. Serum levels of creatinine were assayed by kinetic method (Jaffé method without deproteinization), alanine aminotransferase (ALT) levels were evaluated according to the International Federation of Clinical Chemistry (IFCC) method, and aspartate aminotransferase (AST) levels according to IFCC method without pyridoxal phosphate. Urea and uric acid were measured using urease kinetic method and uricase enzymatic/colorimetric method, respectively. 

Serum levels of triglycerides, total cholesterol, and HDL-cholesterol were assessed using glycerol phosphate oxidase/peroxidase, cholesterol oxidase/peroxidase, and direct colorimetric methods, respectively. These analyses were performed using commercially available kits appropriate for the Automatic Analyser A25 (BioSystems, Barcelona, Spain), according to protocols provided by the kit’s manufacturer (BioSystems, Barcelona, Spain). LDL-cholesterol was calculated using the Friedewald equation [27] and the electrochemiluminescence method assessed thyroid-stimulating hormone (TSH) and free T4 (FT4).

### 2.8. Analysis of Inflammatory, Endothelial Injury, and Oxidative Stress Biomarkers

Blood was harvested into plasma EDTA tubes to determine adiponectin, resistin, leptin, endothelin-1 (ET-1), soluble forms of intercellular cell adhesion molecule-1 (sICAM-1), and vascular cell adhesion molecule-1 (sVCAM-1), tumor necrosis factor-α (TNF-α), interleukin-6 (IL-6) and thiobarbituric acid reactive substances (TBARS) concentrations. Collection tubes were centrifuged on 1000× *g* at 4 °C for 15 min. Plasma was then transferred into cryotubes and stored at −80 °C until analysis. Plasma levels of adiponectin, resistin, leptin, sICAM-1, and sVCAM-1 were assayed by Human Quantikine^®^ Immunoassay kits (R&D Systems, Minneapolis, MN, USA); sensitivities were 0.246 ng/mL, 0.026 ng/mL, 7.8 pg/mL, 0.087 pg/mL, 0.096 ng/mL, and 0.6 ng/mL, respectively. Circulating TNF-α and IL-6 were assessed using Quantikine^®^ High Sensitivity Human Immunoassay kits (R&D Systems, Minneapolis, MN, USA) with sensitivities 0.022 pg/mL and 0.031 pg/mL, respectively. TBARS analysis was performed using Parameter^®^ TBARS Assay (R&D Systems, Minneapolis, MN, USA) with sensitivity of 0.024 µM. Before determining TBARS levels, trichloroacetic acid was added to precipitate proteins and any other interfering substances. Samples were then centrifuged 12,000× *g* at 20 °C for 4 min, and their respective supernatants were transferred into other microtubes and immediately assayed. All assays were performed according to directions provided by the kits’ manufacturer. Intra- and inter-assay coefficients of variation of all analyses were less than 7%. 

### 2.9. Statistical Analysis

Data normality was tested by the Shapiro–Wilk test, and results are expressed as mean ± standard deviation or as median (25th–75th percentile) when appropriate. Unpaired Student *t*-test compared between-group differences for continuous variables and Chi-square test for categorical variables. Pearson´s correlation coefficients or Spearman’s rank were calculated to verify the associations between information obtained by self-report (pre and nadir weight). Statistical analyses were performed using GraphPad^TM^ software (Version 6.0, La Jolla, CA, USA), and the significance level was set at *p ≤* 0.05.

## 3. Results

Demographic/clinical characteristics, bariatric surgery data, and biochemical profile of participants are displayed in Table 1. We should highlight that groups had the same BMI and both were obese. The groups were similar regarding all variables except for neck circumference, fasting glucose, and triglycerides, which were lower in the bariatric vs. non-surgical group (*p <* 0.001). In addition, a higher level of HDL-cholesterol was detected in the bariatric group (*p <* 0.001). Significant correlations were observed between first vs. second self-reports preoperative and nadir weights (*p* = 0.99; *p <* 0.0001).

As displayed in Table 2, hemodynamics and endothelial and microvascular reactivity variables were similar between groups (*p ≥* 0.06). In respect to circulating inflammatory, endothelial injury, and oxidative stress biomarkers, we observed that the bariatric group presented higher levels of adiponectin (Δ: 3.36 ± 2.56 μg/mL, a corresponding difference of ~101.56%; *p <* 0.001) and lower levels of IL-6 in comparison to the non-surgical (Δ: −0.94 ± 0.31 pg/mL, a corresponding difference of ~−26.31%; *p <* 0.001). No significant differences between bariatric and non-surgical groups were found concerning other blood biomarkers. Circulating levels of sICAM-1 were undetected in only one post-bariatric patient.

## 4. Discussion

This study compared the metabolic profile, inflammatory/oxidative status, and endothelial/microvascular functions between post-bariatric patients with high ratios of weight regain vs. non-surgical BMI-, age- and gender-matched controls. Our findings revealed that bariatric patients, even if they are obese, had better clinical indicators linked to body adiposity distribution and metabolic health, expressed as neck circumference, fasting glucose, triglycerides, and HDL-cholesterol than non-surgical ones.

No significant differences between groups concerning hemodynamics, and endothelial and microvascular reactivities, and biomarkers of inflammatory status (leptin, resistin, and TNF-α), endothelial injury (ET-1, sICAM-1, and sVCAM-1), and oxidative stress (TBARS) biomarkers were observed. Of note, on the counterpart, obesity-related inflammatory status was partially reduced in the bariatric group compared to non-surgical controls, reflected by significantly lower IL-6 and higher adiponectin levels. Our data corroborated a previous study describing an inverse relationship between IL-6 and adiponectin levels in patients with obesity before and after diet-induced weight loss [28], which was probably related to IL-6’s inhibition of adiponectin mRNA expression [29].

The beneficial effects of adiponectin on the function of multiple organs and tissues are already known [30], and its positive effects on metabolism reach a wide range of organic sites. In the liver, adiponectin reverses the adverse effects of insulin resistance. It also reduces gluconeogenesis and triglyceride levels in this site, while in skeletal muscle, it upregulates fatty acid oxidation and glucose uptake while decreasing triglyceride synthesis. In the adipose tissue, adiponectin stimulates fat storage in small subcutaneous adipocytes, while in the pancreas, it induces glucose-stimulated insulin secretion and β-cell protection. It also acts on the endothelium, increasing nitric oxide production and decreasing oxidative stress [31]. Besides its metabolic and vasculoprotective actions, adiponectin has anti-inflammatory and antifibrotic activities [32].

Adiponectin secretion seems to be determined more by the quality than by the total mass of adipose tissue [30]. It was previously reported that metabolically healthy adipose tissue expresses greater adiponectin levels than metabolically abnormal (ectopic, unhealthy) ones [33]. However, it is still unclear whether all beneficial metabolic properties of metabolically healthy adipose tissue can be attributed solely to high levels of adiponectin [30].

The observed higher levels of circulating adiponectin and lower IL-6 can explain the improvement of metabolic profile in the bariatric group, reflected by significantly lower fasting glucose and triglycerides levels, greater HDL-cholesterol levels, and maybe also decreased neck circumference. These beneficial metabolic effects of bariatric surgery, even with substantial weight regain, follow a previous study published by our group [24].

It is noteworthy that the metabolic improvements that occurred in a group of patients with obesity (BMI of 40.1 ± 7.7 kg/m^2^), high RWR (58.7 ± 24.3%), and a long time after surgery (10.8 ± 4.7 years). Possibly, our results may be explained by significant weight reduction following surgery (EWL of 84.1 ± 17.9%). The studied patients presented a preoperative BMI of 48.0 ± 6.7 kg/m^2^ with an average body weight change of −49.6 ± 11 kg (ranging from 34 to 76 kg). Our group is similar to a previous prospective cohort of patients with severe obesity that showed prolonged health benefits related to bariatric surgery [1,34]. Another study reported that bariatric surgery contributed to long-term improvements in metabolic status reflected by beneficial changes in plasma glucose and HbA1c and lipid profile [1,2,34].

The microvascular function is determined in part by the functional properties of the endothelium. In obesity, the chronic inflammatory status, insulin resistance, and increased oxidative stress contribute to endothelial dysfunction [35], the earliest marker of atherogenesis [36]. Endothelial dysfunction is characterized by the activation of endothelial cells and consequent upregulation of adhesion molecules on their surface, including ICAM-1 and VCAM-1 [37]. In order to limit vascular inflammation, these adhesion molecules undergo proteolytic cleavage and shedding, increasing their circulating levels [37,38], directly correlated with atherosclerosis [38]. ET-1 is a potent vasoconstrictor released continuously from endothelial cells that contributes to vascular tone regulation [39]. It has been associated with endothelial dysfunction when secreted in higher levels [40] and has been extensively studied as predictor/prognostic marker in coronary artery disease, myocardial infarction, and heart failure [41]. TBARS have been employed as a biomarker of oxidative stress in several models of cardiovascular disease [42]. According to the literature, elevated TBARS levels could predict major cardiovascular events [43] and carotid atherosclerotic plaque progression [44]. In our study, endothelial/microvascular functions were not different between groups. We can speculate that this probably occurred due to similar levels of endothelial injury and oxidative stress biomarkers between groups. Altogether, our data highlight the importance of losing weight on several health biomarkers. Additionally, patients who regain weight may have unfavorable cardiovascular effects, especially those with endothelial dysfunction-related diseases, such as the ones with obesity. Such knowledge is relevant for clinical practice, mainly for the medical and multidisciplinary teams’ approach, i.e., those who work with post-bariatric patients. Bariatric surgery reduces carotid intima-media thickness, improves flow-mediated dilatation-indicators of subclinical atherosclerosis and endothelial dysfunction [10], and decreases cardiovascular morbidity and mortality [11]. Lupoli et al. reported associations between vascular health and better metabolic status, which reflect improvements in visceral obesity, insulin resistance, and dyslipidemia [10]. Although these positive vascular effects seem to occur at the short and long-term follow-ups [10,45,46] and after a loss of excess weight of more than 50% [47], improvements in the vascular function in post-bariatric patients who regained weight were not observed. The return of obesity may explain this finding.

Some limitations of the present study should be mentioned. First, most participants were women, which reflects the population that seeks treatment more often [48]. Second, the study was cross-sectional and causal relations could not be established. Third, we studied only women that underwent a RYGB surgery, the most employed bariatric surgery technique in our country [49], which reflects most of the individuals attended at the Outpatient Care Unit (~89% of female and a proportion of 6.3 post-RYGB patients to 1 post-sleeve gastrectomy) [50]. Hence, our data cannot be extrapolated to other bariatric procedures.

## 5. Conclusions

Despite that we did not find any significant difference between post-bariatric patients with weight recurrence and BMI-matched controls regarding endothelial and microvascular reactivity, our findings suggest that even in those patients with a high ratio of weight regain, the substantial weight loss after bariatric surgery is probably able to promote favorable effects on metabolic and inflammatory profiles, expressed in this study by increased adiponectin and reduced IL-6 levels.

## Figures and Tables

**Table 1:** Demographic/clinical characteristics, bariatric surgery data, and biochemical profile of patients.

Variable	Bariatric Group (*n* = 32)	Non-Surgical Group (*n* = 30)	*p* Value
Demographic characteristics			
Age (years)	44 ± 8	44 ± 11	0.88
Female (n, %)	28 (87.5)	25 (83.3)	0.64
BMI (kg/m^2^)	40.1 ± 7.7	41.0 ± 5.3	0.23
Neck circumference (cm)	36.4 ± 4.4 *	39.1 ± 4.8	0.02
Waist circumference (cm)	109.9 ± 16.2	113.5 ± 12.6	0.34
Hip circumference (cm)	126 [119.0–140.3]	129 [120.0–136.0]	0.84
Clinical history—(*n*, %)			
T2DM	6 (18.8)	10 (33.3)	0.19
Hypertension	13 (40.6)	17 (56.7)	0.20
Dyslipidemia	6 (18.8)	10 (33.3)	0.19
Bariatric surgery data			
Preoperative BMI (kg/m^2^)	48.0 ± 6.7	–	–
EWL (%)	84.1 ± 17.9	–	–
RWR (%)	58.7 ± 24.3	–	–
Nadir weight (kg)	78.5 ± 17.8	–	–
Time since surgery (years)	10.8 ± 4.7	–	–
Metabolic/hormonal profile			
Fasting glucose (mg/dL)	92 [86.5–102.7] *	100 [92.7–131.0]	0.03
HbA1c (%)	5.3 [5.2–5.7]	5.5 [5.2–6.2]	0.06
Total cholesterol (mg/dL)	184.0 ± 46.6	186.7 ± 40.8	0.82
HDL-cholesterol (mg/dL)	58.2 ± 14.0 *	46.7 ± 12.3	<0.001
LDL-cholesterol (mg/dL)	101.0 [78.6–122.9]	107.9 [88.9–134.7]	0.26
Triglycerides (mg/dL)	91.5 [67.2–102.0] *	120 [94.0–145.0]	<0.001
Uric acid (mg/dL)	4.22 ± 1.29	4.77 ± 1.77	0.40
Urea (mg/dL)	31.4 ± 6.9	27.7 ± 9.2	0.15
Creatinine (mg/dL)	0.78 ± 0.20	0.77 ± 0.18	0.81
AST (U/mL)	22.1 ± 7.1	21.8 ± 9.8	0.90
ALT (U/mL)	20.0 [14.5–25.0]	22.0 [14.7–32.7]	0.15
TSH (ng/dL)	3.10 ± 1.89	2.16 ± 1.07	0.06
FT4 (ng/dL)	1.09 ± 0.27	1.13 ± 0.14	0.64

BMI, body mass index; T2D, type 2 diabetes mellitus; RWR, ratio of weight regain; EWL, excess weight loss; HbA1c, glycated hemoglobin type A1c; LDL, low-density lipoprotein; HDL, high-density lipoprotein; ALT, alanine aminotransferase; AST, aspartate aminotransferase; TSH, thyroid-stimulating hormone; FT4, free T4. * *p* value, unpaired Student *t*-test or chi-square test; results expressed as mean ± standard deviation, median [percentiles 25–75], or number (%).

**Table 2:** Hemodynamic parameters, endothelial and microvascular reactivity assessments, and circulating inflammatory, endothelial injury, and oxidative stress biomarkers of the patients.

Variable	Bariatric Group (*n* = 32)	Non-Surgical Group (*n* = 30)	*p* Value
Hemodynamic parameters			
Heart rate (bpm)	63 [58–68]	64 [57–77]	0.52
Systolic BP (mmHg)	126.2 [116.3–136.0]	125.0 [118.5–142.5]	0.58
Diastolic BP (mmHg)	82.0 ± 9.5	77.1 ± 13.1	0.10
Endothelial reactivity by Venous Occlusion Plethysmography			
FBF-bas 1 (mL/min/100 mL)	1.70 ± 0.84	2.07 ± 0.60	0.06
FBF-hyper (mL/min/100 mL)	7.10 [5.84–10.45]	8.94 [6.43–10.29]	0.52
FBF-bas 2 (mL/min/100 mL)	1.26 [0.92–1.60]	1.55 [1.19–1.90]	0.13
FBF-nitro (mL/min/100 mL)	1.38 [1.12–1.82]	1.49 [1.22–1.79]	0.34
Microvascular reactivity by Laser Speckle Contrast Imaging			
Baseline flow (APU)	35.40 ± 11.83	35.36 ± 10.36	0.98
Peak flow during PORH (APU)	76.69 ± 22.14	77.78 ± 18.58	0.83
Post-occlusive flow (APU)	34.79 ± 15.31	35.18 ± 10.75	0.90
Duration of PORH (s)	0.51 [0.38–0.90]	0.46 [0.31–0.55]	0.25
Blood biomarkers			
Adiponectin (μg/mL)	6.07 [3.97–8.61] *	3.10 [2.50–3.78]	<0.001
Leptin (ng/mL)	51.38 ± 23.76	49.05 ± 20.45	0.68
Resistin (ng/mL)	8.48 ± 2.90	7.40 ± 2.76	0.14
IL-6 (pg/mL)	2.02 [1.26–2.93] *	3.00 [2.34–4.06]	<0.001
TNF-α (pg/mL)	0.769 [0.636–1.034]	0.834 [0.730–1.053]	0.56
ET-1 (pg/mL)	1.42 [1.24–1.76]	1.31 [1.16–1.55]	0.20
sICAM-1 (ng/mL)	982.2 ± 278.2	934.6 ± 268.2	0.51
sVCAM-1 (ng/mL)	634.5 [559.5–709.3]	591.9 [498.5–666.2]	0.09
TBARS (µM)	1.129 [0.957–1.304]	1.073 [0.984–1.294]	0.86

BP, blood pressure; FBF, forearm blood flow; FBF-bas 1, FBF at baseline flow 1; FBF-hyper, peak FBF during reactive hyperemia; FBF-bas 2, FBF at baseline flow 2; FBF-nitro, FBF after sublingual nitroglycerin administration; PORH, post-occlusive reactive hyperemia; APU, arbitrary perfusion units; IL-6, interleukin-6; TNF-α, tumoral necrosis factor alpha; ET-1, endothelin-1; sICAM-1, soluble intercellular adhesion molecule-1; sVCAM-1, soluble vascular cell adhesion molecule-1; TBARS, thiobarbituric acid reactive substances. * *p* value, unpaired Student *t*-test; results expressed as mean ± standard deviation or median [percentiles 25–75].

## Data Availability

The datasets used to support the findings of this study are available from the corresponding author upon request.
